# Lecture on Practical Education in Medicine, and on the Course of Instruction at the N. Y. Hospital

**Published:** 1847-03

**Authors:** John Watson

**Affiliations:** One of the surgeons of that Institution


ART. III.—*Lecture on practical Education in Medicine, and on the course of instruction at the N. Y. Hospital. Delivered at the Hospital, Nov. 3d,* 1846; *by* JOHN WATSON, M. D., *one of the surgeons of that Institution.*

The lecture sessions at most Medical Institutions being now in progress we begin to receive published introductories from various quarters. The custom of preparing these discourses for publication or, perhaps, we should rather say the habit of publishing them appears to us a good one. Besides being voices from different schools, giving some indications of their condition and prospects, they relate generally to topics in which the profession at large have, or should have an interest. They constitute an important portion of American periodical literature. For one, we are always glad to receive them, and in our capacity of Journalist should oftener make it a point to notice them at length, were it not that they are generally pretty extensively circulated throughout the country.

The address the title of which we have given above was delivered the opening of a course of clinical instruction of the N. Y. Hospital. rofession will be gratified to learn that the officers of that valuable institution have resolved upon an extensive and systematic plan of clinical instruction; and that it is contemplated to make it hereafter, in all respects a practical school of medicine. All large Hospitals should embrace this among their humane provisions. Next to the relief of those actually suffering from disease, come the necessity and duty of making the knowledge derived from the study of disease in public charities available to mankind. In this way society is repaid, and with interest, for all that it bestows upon these charities. Indeed, putting out of the question the obligation of affording relief to the sick and needy (which of course, we have no disposition to depreciate) it is obvious that no better investment of public bounty can exist than in Hospitals and Dispensaries, provided they are made subsidiary to the increase and diffusion of Medical and Surgical knowledge. Charity is here, not figuratively but literally, and emphatically twice blest. While it relieves the sufferings of those who are unable to procure the comforts and attentions which illness requires, it benefits, also, those who can avail themselves of everything that health and friendship can command, in the greater professional experience and skill which it places at their disposal. If legislators and communities would take this correct, and, to them, important view of the subject, there would not be so much difficulty in procuring means for the endowment and support of those institutions : still less would it ever be the case that a narrow minded policy would attempt to place restrictions upon their application to purposes of medical instruction.

The address of Dr. Watson is precisely what would be looked for from that source, containing sound, practical precepts, conveyed in an impressive but unostentatious style.

The following quotation presents views which the student would do well to consider, and act upon, before assuming the responsibilities of practice:

The practice of medicine is an art, as strictly and as literally such, as the art of building, of printing, or of metal working; and the more our systems of education are made to conform to this view of the matter, provided the principles of this art are not in the meanwhile neglected, the better they will subserve the purposes of the profession. The joiner, the shipwright, and the smith, initiate their youth into the magic uses of the saw, the hammer, and the file; and they would laugh at the philosopher who should attempt to convince them that these youths, by studying the principles of mechanics, of the wheel-axle, of the lever, the screw, or the inclined plane, as taught in books and lecture rooms, might be saved from the disgreeable necessity of plying their bones and muscles in the workshop.

Like those engaged in the study of other pursuits, the student of medicine must be taught to exert his own powers; and before entering upon the responsible duties of his profession, he should be made to give evidence of practical acquirement. For, without this, his principles will serve him to but sorry purpose at the bed-side. They may impart to him the semblance of knowledge; they may serve him in discourse; they may enable him to pass, even with some eclat, through the ordeal of a green-room examination. But unsupported and unenlivened by personal observation and experience, they must gradually escape him; or, in the long run, serve only to mislead him by the flattering, though false, conviction of being really master of all that he remembers.

We quote his views respecting the progressive improvement of our Medical Schools, and the fact that policy as well as propriety dictates still farther advancement. In the latter we do not doubt he is correct.

"The course of education in our well appointed colleges—and especially in the rival schools of this city (rivals be it ever hoped only in their ability for usefulness)—is every year becoming more and more elevated. The time, I trust will soon come when these useful institutions may rise above the obstacles that still prevent them from placing their curriculum of study on a level with that of the most reputable schools of Europe.

And let it not be said (for the charge is a libel on the people of this nation) that we are not prepared for such a system. Are our people less intelligent than those of other nations? Are they less abundantly supplied with the conveniences, the comforts, or even the luxuries of life? Are they less disposed to patronize talent and acquirement? I speak not of the residents of our larger cities, I speak of our people as a nation. Gentlemen, the medical institution which first comes up to the reasonable demands of the profession in this respect, and, possessing the facilities, requires of its graduates practical acquirements equal to those at present enjoined upon the students of European schools, will find its interest in the measure. We hear, in all quarters, loud and lamentable outcries against the reign of quackery and imposture. But the rifest and rankest quackery of this land, is the quackery of half-educated graduates in medicine."

We give the two concluding paragraphs of the discourse, being portions of advice to pupils designing to avail themselves of the advantages of the Hospital. The last paragraph is particularly gratifying, coming, as the advice does, from so eminent an operative surgeon as Dr. Watson.

"Do not fall into the puerile fondness of hunting after strange sights, rare diseases, and great cases. The dressing of an ulcer, the setting of a bone, the treatment of a pleurisy, pneumonia, or fever,—these, and such as these, should be your study here. Striking cases you may look at ; but the business that should fix your attention, is that which best prepares you for the daily business of the profession.

"Finally avoid that morbid appetite for surgical operations, so long magnified and so much over-rated. Surgery is a good thing, a useful, an excellent thing in its way; but too much of it is a great evil. And the sooner you find out this for yourselves, the better for your patients."

